# Development of Subjective Well-Being in Adolescence

**DOI:** 10.3390/ijerph16193690

**Published:** 2019-09-30

**Authors:** Ricarda Steinmayr, Linda Wirthwein, Laura Modler, Margaret M. Barry

**Affiliations:** 1 Department Educational Sciences, Psychology, and Sociology, TU Dortmund, Emil-Figge-Str. 50, Dortmund 44227, Germany; Linda.Wirthwein@tu-dortmund.de (L.W.); Laura.Modler@tu-dortmund.de (L.M.); 2 Health Promotion Research Centre, National University of Ireland Galway, University Road, Galway H91 TK33, Ireland; margaret.barry@nuigalway.ie

**Keywords:** subjective well-being, personality, parental engagement, parental expectations, intelligence, gender, socio-economic status, academic achievement

## Abstract

Despite the importance of subjective well-being (SWB) for students’ mental and physical health, there is a lack of longitudinal studies investigating the development of SWB in adolescents and what factors are associated with it over time. The present study seeks to shed further light on this question by investigating adolescents longitudinally. A sample of German academic tracks students (*N* = 476) from five schools were followed longitudinally over a time period of 30 months with four measurement points from Grade 11 to Grade 13. Alongside the longitudinal assessment of SWB (mood and life satisfaction), a range of other factors were also assessed at t1 including; demographic factors (sex, age, socio-economic status (HISEI)), intelligence, grades (report cards provided by the schools), personality (neuroticism, extraversion) and perceived parental expectations and support. Latent growth curve models were conducted to investigate the development of SWB and its correlates. On average, mood and life satisfaction improved at the end of mandatory schooling. However, students significantly differed in this pattern of change. Students’ life satisfaction developed more positively if students had good grades at t1. Furthermore, even though introverted students started with lower life satisfaction at t1, extraverts’ life showed greater increases over time. Changes in mood were associated with socio-economic background; the higher the HISEI the more positive the change. As social comparisons in school performance are almost inevitable, schools should intervene to buffer the influence of school grades on students’ SWB.

## 1. Introduction

Countless studies on Subjective Well-Being (SWB) have demonstrated the importance of SWB for several important outcomes in adulthood such as, mental health [1], survival [2], disease progression and recovery [3], cardiovascular functioning [4], health behavior [5,6] and vocational success [7,8]. Given the importance of SWB in adults it is not surprising that SWB has recently also received a lot of attention in adolescents and children [9]. Comparable to the results found for adults, several studies also demonstrated associations between SWB in children and adolescents and important outcomes such as health behavior [10], mental health [11], and academic achievement [12]. As SWB seems to be a potent predictor of several important outcomes, it is imperative to understand how SWB develops and the factors associated with its development [13]. Only a few studies have investigated changes in the SWB of adolescents focusing on different age groups [14,15] and just very few investigated whether adolescents differ in their SWB development depending on variables such as socio-demographics or personality. Therefore, despite the importance of SWB for students’ mental and physical health, there is a lack of longitudinal studies investigating the intraindividual development of SWB in adolescents and what factors are associated with it. The present study seeks to shed further light on this question by investigating the intraindividual development of SWB in adolescents longitudinally.

### 1.1. Subjective Well-Being of Adolescents

There is increasing recognition of the importance of children’s and adolescents’ social and emotional development and the need to create supportive conditions to enable students’ optimal academic, emotional and social functioning [16,17]. In this regard, SWB is a central construct examined in the context of positive psychology and mental health promotion [18]. Compared to the research on adults’ SWB, the SWB of children and adolescents has received comparatively less attention [19,20]. Similar to the research on adults’ SWB, the well-being of children and adolescents has been defined in various ways.

Generally, two theoretical approaches can be differentiated which have an impact on the conceptualization and operationalization of SWB [21,22]. The hedonistic perspective defines SWB as the presence of joy or happiness. In this context, SWB comprises individuals’ cognitive evaluations of their lives as a whole (i.e., global life satisfaction) and reports on affective well-being such as pleasant and unpleasant emotions [23]. Compared to affective components, life satisfaction is known as the most stable cognitive component of SWB and is analyzed more frequently than the affective components [24]. According to the eudaimonic perspective, well-being occurs when an individual lives in congruence with his or her subjective beliefs and values. According to this approach, SWB is measured multidimensionally [25]. Depending on the underlying theoretical model, different variables are used to define eudaimonic SWB. Since the factorial structure of these multidimensional models could not be confirmed continuously [26] and because of high intercorrelations between indicators and determinants of eudaimonic well-being components, we refer to the hedonistic perspective of SWB in the current publication. Furthermore, the use of the three SWB indicators, life satisfaction, positive and negative affect, has become well-established in the scientific literature [27]. Hence, in the following, we primarily refer to studies that used this definition of SWB.

### 1.2. Changes in Subjective Well-Being over Time During Adolescence

Generally, SWB and in particular life satisfaction appears to be comparatively stable over time [28], although some life events might have an influence on its stability [29]. Theoretical models refer to bio-social-cognitive approaches [30,31] which explain individual differences in SWB by focusing on different personality variables as well as on environmental factors (e.g., life events). Whereas several studies focus on the development of adults’ SWB [27], the important period from adolescence to young adulthood has received less attention so far [32]. This is surprising, given the important changes during this period of time, including physical, neurodevelopmental and psychosocial changes [33]. Most research on adolescents focuses on the cognitive component of SWB (i.e., life satisfaction) while the affective component has rarely been examined. In the last Programme for International Student Assessment (PISA), life satisfaction was assessed and in this cross-sectional study adolescents were, overall, satisfied with their global life in Western Countries, even though there were some differences between countries [16].

Empirical results from longitudinal studies focusing on changes in SWB throughout childhood and adolescence are still scarce. Salmela-Aro and Tuominen-Soini [14] addressed the development of life satisfaction from middle (15 years) to late adolescence (17 years) during the transition from comprehensive school to upper secondary school (four measurement points during two years) in Finland. Results showed an intraindividual increase of life satisfaction. Moreover, significant individual variation in both the initial level of, and intraindividual changes in, life satisfaction was found, but just for girls: the lower the initial level of life satisfaction, the higher the increase. For boys, there also was an increase in life satisfaction during this transition, but there was no significant variation regarding the linear change. Other studies focusing on younger adolescents found a decrease from early (about 13 years) to middle adolescence (about 15 years) in cognitive as well as in affective SWB components [15,19,34]. This decline was also found in several cross-sectional cohort studies focusing on affective as well as cognitive indicators of SWB in adolescents between ten and 16 years [14,35,36]. Given these empirical results, it might be relevant to focus especially on the respective age of the adolescents investigated in each study. In this context, it has to be kept in mind that the period of adolescence lasts up to about ten years and, hence, it may be important to differentiate between early, middle, and late adolescence [14]. In summary, little is known about the longitudinal change in cognitive and affective SWB during late adolescence, as most studies in this age range used a cross-sectional design and apart from the study by Salmela-Aro and Tuominen-Soini [14], no study has investigated longitudinally whether the decline in SWB is reversed in late adolescents. The present study addresses not only the changes in SWB over time in late adolescents, but also whether some variables moderate the change in SWB, i.e., whether change in SWB is more or less the same among all adolescents or if there are interindividual differences in the intraindividual change that are explained by further variables. To identify possible moderators of these changes, in this study we consider the most important variables associated with SWB in adolescence.

### 1.3. Correlates and Determinants of Subjective-Well-Being

Many studies have been dedicated to examining the determinants and correlates of children’s or adolescents’ SWB [37]. The research on determinants of SWB has focused on “bottom-up” variables (such as demographics) and “top-down” variables (such as personality) [23,27], with top-down variables (personality but also social relationships) being especially relevant for children’s and adolescents’ SWB [38,39]. Moreover, the family and school environment has been shown to be important [40]. In this context, ecological system theories assume an impact of family, school, and other layers of the environment on children’s and adolescents’ positive development [41]. Schools are believed to have a particularly important influence as an environment that contributes to the SWB of adolescents [42]. Hence, a multitude of different bottom-up and top-down variables as determinants or correlates of SWB have to be taken into account. However, there is a lack of studies investigating different correlates or determinants simultaneously so that little is known about their incremental validity independent from each other [12]. In the following, we refer to studies that focus on the most relevant correlates of SWB.

Among the personality variables, extraversion and neuroticism show substantial associations with cognitive and affective well-being [39,43,44,45,46]. Extraversion is a personality trait characterized by high sociability and high activity levels whereas neuroticism refers to a trait characterized by negative emotions such as fear, anxiety, or worry [47]. In a study with adolescents (aged between 13 to 19 years), neuroticism (*r* = −0.65), and extraversion (*r* = 0.30) showed the highest correlations with life satisfaction among the Big Five factors of personality [48]. With regard to the affective facets of SWB, the correlations are comparable in size [45].

An important school variable in the context of adolescents’ SWB is academic achievement [49,50,51]. There appears to be comparatively high pressure on students to succeed in school because in our society success at school is more decisive than ever for opportunities in the further course of education and in professional life [52]. Good school performance could, therefore, have a positive impact on SWB, whereas poor school performance could have a negative impact. This is consistent with studies showing that good school performance serves as a protective factor against mental disorders [53,54]. Research has demonstrated that there are substantial associations between both constructs: The meta-analysis by Bücker et al. [55] established a correlation of *r* = 0.16 between academic achievement and SWB. Descriptively, there are higher positive correlations of life satisfaction with school achievement (*r* = 0.20) than with the affective components of well-being (*r* = 0.16). Longitudinal studies on the associations between SWB and academic achievement are still scarce. Steinmayr et al. [12,56] undertook a longitudinal investigation of the relationship between academic achievement (GPA) and cognitive as well as affective well-being in a sample of adolescents (at the age of 16 years). It was shown that school grades can have an effect on the changes in cognitive well-being (but not affective well-being), however, there was no reverse effect during a period of one year. Ng, Huebner and Hills [57] found a small reciprocal effect between life satisfaction and grades in older adolescents.

General intelligence is highly correlated with academic achievement [58,59]. However, intelligence as a determinant or correlate of SWB has received comparable less attention so far [60]. Diener [61] assumed that intelligence might be a relevant predictor for SWB because it is an important resource in our society. Probably due to different operationalizations of both SWB and intelligence, correlational analyses found extremely heterogeneous associations between both constructs (ranging from *r* = 0.44 to *r* = −0.08 [50,62]). The majority of studies found negligible or not significant correlations [60].

Gender and socio-economic status are important socio-demographic variables frequently investigated in both educational and SWB research [63,64]. Gender differences regarding SWB are rather small in adults [65]. Focusing on adolescents, the results are heterogeneous but seem to be small in magnitude as well (*d* < 0.30) [66,67,68]. Salmela-Aro and Tuominen-Soini [14] demonstrated in a sample of late adolescents that girls’ life satisfaction was initially lower but increased more strongly, nearly reaching the life satisfaction level of boys at the age of 17. However, the authors did not examine whether the change in SWB was significantly different for boys and girls. Socio-economic status (SES) is usually operationalized by parents’ education, income, as well as their occupation [64,69]. Studies investigating the relevance of SES in the context of adolescents’ SWB have shown divergent results. Many studies have not detected a relationship between both variables [63,70], whereas other studies have established a positive association [71]. However, all studies have only investigated a linear relationship between SWB and SES and gender, respectively. We are not aware of any study that has investigated whether SES has an impact on the intraindividual change in SWB in adolescents, i.e., if SES is an important predictor or correlate of positive change in SWB. As students in late adolescence normally have no income of their own, and as economic resources are needed to continue one’s academic career, it may be that, especially at that age, SES impacts on the intraindividual changes in SWB.

The association between social support and SWB is well documented in the literature [72]. Peer support especially but also familial support seem to be important predictors of adolescents’ SWB [73]. Generally, it is assumed that social support empowers individuals to cope better with negative life events [74]. In this context, family relationships (e.g., parent-child relationships) as well as the specific parenting behavior show substantial associations with adolescents’ SWB [75]. In a study by Garcia et al. [76] focusing on adolescents from different countries especially parental warmth and low parental strictness were associated with personal as well as social well-being (operationalized as self-esteem in different domains). A recent meta-analysis found that perceived social support, in particular, shows positive associations with cognitive and affective facets of SWB [77]. As adolescents spend most of their time in school, perceived parental involvement regarding school and parents’ values regarding scholastic attainment may also be an important resource of adolescents’ SWB [78]. So far, parental involvement and support regarding school as well as parental values have predominantly been investigated in the context of academic achievement [79]. Results from PISA have shown that spending time talking to one’s children about school seem to be a particularly important correlate of life satisfaction [9]. However, to our knowledge, no previous study has investigated this relationship longitudinally.

Only a few cross-sectional studies have analyzed several different correlates of adolescents’ SWB simultaneously to test whether they possess incremental validity in predicting SWB independent from and beyond each other. Trzcinski and Holst [32] have analyzed the life satisfaction of adolescents (17 years old) from the German Socio-Economic Panel (SOEP), a representative study of German households. Different demographic and socio-economic characteristics, as well as different individual variables (e.g., personal control) and variables assessing the relationship with parents and peers were measured. Similar to the results found with adults, personal relationships, but also individual variables, were relevant for life satisfaction, whereas demographic variables were less relevant. Steinmayr et al. [12] focused on the relevance of different school variables such as school climate, different motivational variables, and grades for cognitive and affective well-being in a sample of *N* = 767 Eighth- and Ninth-Grade students. Results showed that a positive school climate as well as self-efficacy, the worry component of test anxiety, and grades were correlated with SWB components.

### 1.4. Change in Subjective Well-Being: Moderators

Previous studies have frequently investigated whether SWB is stable or unstable during different periods of life. Research regarding other psychological constructs relevant for the development of adolescents such as mental health or self-esteem have shown that trajectories can be diverse for different groups of adolescents [14,80,81]. Hence, several moderators might have an impact on individual trajectories. Apart from the study by Salmela-Aro and Tuominen-Soini [14,82], studies investigating factors that are associated with change in SWB in the period of late adolescence are very scarce. The authors examined whether academic achievement (self-reported grades), socio-economic status, educational aspirations and self-esteem predicted the level and changes in life satisfaction. Among these variables, academic achievement and self-esteem were especially important for changes in life satisfaction. Furthermore, they found that the trajectories of life satisfaction seem to be more heterogeneous for girls than for boys. Using a different methodological approach but with the same sample of students, Salmela-Aro and Tynkkynen [83] reported that students with high academic achievement and boys were overrepresented in a group with a high stability of life satisfaction during the transition to post-compulsory education. Focusing on younger students, Gonzalez-Carrasco et al. [19] investigated a sample of *N* = 940 Spanish adolescents (age: *M* = 12.02; *SD* = 1.5) twice within one year. A decrease in different cognitive and affective measures of SWB was found and the decrease was higher for girls than for boys. Shek and Liu [40] analyzed *N* = 3328 Chinese adolescents (age: *M* = 12.59; *SD* = 0.74) over a period of six years. Several variables were found to be associated with a decline in life satisfaction: although boys showed a higher level of life satisfaction than girls at the first measurement point, they had a faster decline than girls. Furthermore, adolescents with a higher positive identity and spirituality had a faster decline. Moreover, adolescents with a good relationship with their mother also displayed a higher life satisfaction, but showed a higher decrease across the years. Another variable associated with changes in SWB seems to be intelligence. There are some indications that general intelligence might have an impact on the variability in life satisfaction: controlling for several demographic variables, the higher the level of intelligence, the more stable levels of life satisfaction were found to be in a large cohort of British adults [60]. The previously mentioned studies show that different individual as well as demographic variables may be relevant for changes in SWB. However, so far, no study has investigated whether different bottom-up and top-down variables simultaneously are relevant for changes in SWB. Moreover, the period of late adolescence has received little attention in this respect, and the affective component of SWB has rarely been investigated.

Besides individual variables different familial variables such as the parenting style, parental engagement, and the quality of parent-adolescent relationships have effects regarding the change in SWB [76,83]. In summary, children and adolescents show an increase in SWB when they experience more support and involvement from their parents [83].

Taken together, and in keeping with the results found in adults, SWB appears to more strongly associated with personality traits, individual variables (top-down factors) or familial variables and social support than with socio-demographic variables (bottom-up factors). Although there are some studies which have examined individual, psychosocial, or demographic determinants of adolescents’ SWB [43,45], most studies are cross-sectional [32] and there is a lack of longitudinal research focusing on the intraindividual development of adolescents’ SWB and the factors that are associated with its development over time. Furthermore, different bottom-up as well as top-down variables have rarely been examined simultaneously [14]. The period of late adolescence especially has received little attention so far. Hence, the current study seeks to shed further light on the development of adolescents’ SWB and to identify what factors are associated with it by examining adolescents longitudinally. The aim of our study was to investigate the overall change in SWB (cognitive as well as affective facets), the interindividual difference in that change and what variables are associated with SWB and its change. Therefore, we refer to the most relevant bottom-up variables (socio-economic status, gender, grades) as well as to top-down variables (extraversion and neuroticism, intelligence) and family variables (parental engagement and expectations). Given the results reported by Salmela-Aro and Tuominen-Soini [14], we expect a positive development of life satisfaction over time. The question of whether this increase also applies to the emotional SWB facet is also examined. Furthermore, we investigate several moderators of the intraindividual change in SWB. Additionally, drawing on the results by Salmela-Aro and Tuominen-Soini [14], as well as those by Shek and Liu [40], we expect gender and academic achievement to moderate the intraindividual change in life satisfaction. We had no specific expectations concerning mood and all other moderators.

## 2. Materials and Methods

### 2.1. Sample

The data are part of a 3-year longitudinal project on students’ academic development at the end of secondary school that was conducted between 2008 and 2011 [84]. The project covered 4 measurement points and 476 German students (232 boys and 244 girls) from five academic-track schools (Gymnasium) in North-Rhine Westphalia (2 schools) and Baden-Württemberg (3 schools). In 2010, about 32% of the student population in Germany graduated from this kind of school (Federal Statistical Office, 2011). At measurement point 1, students were in 11th Grade and on average 16.43 (*SD* = 0.55) years old. Attending a school at that grade is voluntarily as schooling is only compulsory for 10 years in Germany. The mean highest occupational index of parents’ occupations was *M* = 56.52 (*SD* = 12.72) and thus above the average HISEI found in a representative student population of approximately the same age and cohort (Average HISEI for Germany, PISA 2009; *M* = 48.9, *SD* = 15.6) (p. 235, [85,86]). An above average HISEI of this magnitude is typical for this kind of school [84] (p. 147). Participation was voluntary and parents’ full informed consent was required before students could participate. As the project was part of the schools’ job and application training program, nearly 100% of the basic population participated at one or more measurement occasions in the project.

### 2.2. Procedure

The project started at the beginning of Grade 11 (t1, 2008). The second measurement occasion (t2, 2009) took place when students were at the beginning of the second term in Grade 11. The last two measurement occasions followed at an interval of 1 year at the beginning of the second term in Grade 12 (t3, 2010) and Grade 13 (t4, 2011). Besides other variables that were assessed in the longitudinal project, the variables of interest were assessed by trained research assistants during regular classes in students’ classrooms in groups of about 20 students. At t1, students first answered questions on their age, gender, families, and social background, followed by questionnaires assessing, among other variables, their personality. After working on the self-report questionnaires, students filled in the test on their cognitive abilities. Follow-up was also assessed by trained research assistants. Not all variables were assessed at all measurement points due to time restrictions. At all measurement points students filled in the questionnaire on their life satisfaction. The questionnaire on the second component of subjective well-being (mood) was only filled in at measurement points 2 to 4. All other variable considered in the present study were assessed at measurement point 1. Table 1 gives an overview of when each of the variables in the present study was assessed. Data of the different measurement occasions and report cards (see below) were matched based on a code that only the students could create and totally guaranteed their anonymity. We verify that the study is in accordance with established ethical guidelines. Approval by an ethics committee was not required as per the institution’s guidelines and applicable regulations in the federal state where the study was conducted.

### 2.3. Instruments

#### 2.3.1. Subjective Well-Being (SWB)

In this study, SWB was measured using the Habitual SWB Scale (HSWBS) [87], whereby it is possible to survey the cognitive as well as the affective dimension of subjective well-being. The original scale consists of a mood-level scale [88] (six items) and a satisfaction with life scale [89] (seven items), both of which are designed to measure SWB [90].Three items of the life satisfaction scale assess how satisfied one is with one’s life in the present (e.g., “I am satisfied with my life”), two items assess the satisfaction with life in the past (e.g., “When I look back on my life so far, I am satisfied”) and the last two items are about the expectations for one’s life in the future (e.g., “I believe that much of what I hope for will be fulfilled”). The mood-level scale is a German short version of the Mood-Level Scale by Underwood and Froming [91]. This scale assesses the affective component of SWB by evaluating the experience of positive and the absence of negative emotions (e.g., “Mostly I am happy”). In this study, we used a shortened form from five items instead of the original six items form. However, the internal consistency of the mood-level scale (*α* < 0.70) and of the life satisfaction scale (*α* < 0.80) are comparable to those by Dalbert [87].

#### 2.3.2. Socio-Economic Status

Socio-economic status was assessed by the International Socio-Economic Index of Occupational Status (ISEI), considering just the highest index in the family (HISEI) [9]. The HISEI is calculated on the basis of students’ open-ended statements about their parents’ occupation. The reported occupations are then matched with a widely used standard International Socio-Economic Index of Occupational Status, which takes into account the required level of education and the income distribution for different occupations (ISEI) [92]. As noted previously, the distribution of occupational prestige in our sample did not match nationally representative data [83], but did match those for samples attending the same school type [86].

#### 2.3.3. Intelligence

The Intelligence Structure Test 2000 R (IST) [93] was applied to assess intelligence. This multidimensional intelligence measure covers nine tasks in the basic module, by which it is possible to create three primary factors (verbal, numeric and figural reasoning) as well as a general reasoning factor. As reasoning is very closely related to general intelligence [94], reasoning can be used as a proxy of ‘general intelligence’ or g [95].

#### 2.3.4. Personality

The NEO Five-Factor Inventory (NEO-FFI) evaluates the five basic personality factors Neuroticism, Extraversion, Openness to Experience, Agreeableness and Conscientiousness [96]. It consists of 60 items, divided into 12 items for each trait. In the present study, personality was assessed with the German version of the NEO-FFI [97]. As the meta-analysis by Steel and colleagues [46] demonstrated that Neuroticism and Extraversion were strongly associated with SWB, only these two scales were considered in the present study (Neuroticism: *α* = 0.86; Extraversion: *α* = 0.80).

#### 2.3.5. Gender

Gender was coded with 1 = female students and 2 = male students.

#### 2.3.6. Academic Achievement

At all measurement times academic achievement was measured with Grade Point Average (GPA) as indicated by students’ report cards at t1. Report cards were delivered by the schools, coded and anonymized and then matched with the other measures. In Germany, the grades range from “1” (outstanding achievement) to “6” (poorest achievement). For the purpose of analysis, grades were inverted so that higher scores represented a better performance.

#### 2.3.7. Parental Involvement Regarding School and Parental Scholastic Values

In order to assess parental involvement with school and the quality of students’ relationships with their parents concerning school, we asked students to rate on a 5-point Likert scale; “How often does a family member help you with your work for school?” (support) and “How often do you talk with your parents about school” (indicator of the quality of relationship with parents concerning school) (1 “never”, 2 “rarely”, 3 “sometimes”, 4 “often”, 5 “very often”) [78,98]. To assess parental scholastic values, we asked students to rate “How much do your parents value high scholastic attainment?” (1 “very little”, 2 “little”, 3 “average”, 4 “quite some”, 5 “a lot”). This item is based on the parents’ questionnaire used in a study by Galper, Wigfield, and Seefeldt [99] (p. 901). All items were considered separately as they were not correlated highly.

### 2.4. Analyses

As in all longitudinal studies, sample attrition occurs and needs to be taken into account. At t1 *n* = 421 students participated, t2: 416, t3: 320 and t4: 289. The decline in participation rate after t2 is due to the drop out of one school in Baden-Württemberg. The reasons for drop-out were not related to the project. We used the Full Information Maximum Likelihood (FIML) [100] algorithm for handling missing data.

In order to test for mean level changes over time in both components of SWB, latent growth modelling (LGM) procedures were run using AMOS 25.0 (IBM, Armonk, NY, USA). We chose LGM for several reasons. First, LGMs have been found to have more power in detecting changes over time than repeated measurement analysis of variance [101]. Second, when using LGM missing data can be handled by FIML. Third, applying LGM allows for a description of both an overall pattern of change in the construct of interest over time and individual differences in these trajectories [102]. A simple latent growth curve model (LGCM) consists of a Slope and an Intercept Factor. The Slope factor refers to the rate of change and describes the direction of the development. The mean of the Slope factor and its algebraic sign indicate the overall magnitude of the change and its direction, whereas its variance indicates the interindividual differences in change. Another important component of a LGCM is the Intercept Factor. The mean of the Intercept factor describes the average initial value of a construct, mostly the first measurement within a longitudinal investigation. Both interindividual and intraindividual differences in changes over time are considered [103]. Therefore, it is not only possible to test if there is an average change over time (i.e., that the slope factor is significantly different from zero) but also if individuals differ in that change (i.e., slope factor’s variance is significantly different from zero) and what variables are associated with those interindividual differences in the intraindividual change.

Two LGMs (life satisfaction and mood) were set up with four and three measurement occasions respectively, as well as a correlated latent intercept and slope factor to test whether mood and life satisfaction change over time. Loadings of all measurement occasions on the Intercept Factor were set to 1. Loadings of the first measurement occasion on the Slope factor were set to 0, whereas the loading of the last assessment occasion was fixed to 1. As we did not have any hypothesis concerning the shape of the development (e.g., a linear trend), the loadings of the remaining measurement occasions were freed. Furthermore, we constrained constructs’ variance to be equal across measurement points. We did not consider the nested structure of the data as students changed courses during our study after 11th Grade. In the next step we set up LGMs with one covariate at a time to test which of the investigated covariates are associated either with the intercept and/or the slope factors. We only considered one covariate at a time to test which covariate on its own is associated with interindividual or intraindividual differences in mood and life satisfaction. Finally, we set up a Structural Equation Model (SEM) in which we regressed each Intercept and Slope factor simultaneously on all covariates. Similar to the method of structural equation modelling, different fit indices provide information on how good the LGCM fits to the underlying data. Because of their widespread use [104], we refer to the comparative fit index (CFI), the root-mean square error of approximation (RMSEA) along with its associated confidence intervals, and the chi-square test statistic to evaluate goodness of fit of the tested models. CFI values greater than 0.90 are typically assumed to reflect an acceptable fit to the data; Hu and Bentler [105] suggest that values for RMSEA should be smaller than 0.06, Browne and Cudeck [106] consider that values up to a RMSEA ≤ 0.05 indicate a very good model fit and that a RMSEA ≤ 0.09 is still an indicator for an acceptable error of approximation.

## 3. Results

### 3.1. Descriptives

Table 2 depicts means, standard deviations and intercorrelations of all scales. Means and standard deviations of all scales are in the expected range. The demographic variables HISEI and gender were neither correlated with life satisfaction nor with mood at all measurement points. Intelligence was never associated with mood but displayed a small correlation with life satisfaction at t4. The investigated personality traits, neuroticism and extraversion, correlated with both mood and life satisfaction at all measurement points representing medium to large effects. How often a family member helps doing tasks for school was neither associated with life satisfaction nor with mood at all measurement points with one exception (t4: mood). How often parents talk with their children about school was associated with life satisfaction in the first year of the study (t1 and t2) and with mood at t2 and t4. Grades at measurement point 1 were associated with life satisfaction at all measurement points but not with mood.

### 3.2. General Developmental Model

The model for life satisfaction provided the following fit criteria: *χ^2^* (6) = 14.55; *p* = 0.02; CFI = 0.99; RMSEA = 0.06. The model for mood yielded the following fit indices: *χ^2^* (1) = 9.64; *p* = 0.01; CFI = 0.97; RMSEA = 0.09. A comparison with the above outlined guidelines indicated that all models showed at least an acceptable fit to the data.

First, we inspected the means of the different Slope factors indicating the average magnitude and direction of change. All means were significantly different from zero and all means were positive, i.e., students’ SWB in general underwent a change for the better (life satisfaction: *M* = 0.16, *SE* = 0.05, *p* < 0.01; mood: *M* =0.12, *SE* = 0.06, *p* = 0.05). The factor loadings on all slope factors, that represent the proportion of change relative to the total change occurring over all time points, reflected an overall nonlinear increase in both constructs (cf. Table 3). Life satisfaction did not change significantly from t1 to t2 but did so from t2 to t3 and from t3 to t4. More significant changes in mood were found from t2 to t3 compared to the changes from t3 to t4.

In the next step, we investigated whether these observed declines applied to all students (variance not different from zero) or whether there were significant interindividual differences in change (variance different from zero). The variances of the Slope factor of all constructs were significantly different from zero (life satisfaction: *s^2^* = 0.15 (*SE* = 0.07), *p* = 0.04; mood: *s^2^* = 0.20 (*SE* = 0.10), *p* = 0.05) indicating that there were substantial individual differences among students considering their increase in both constructs.

Together, these findings were in line with our assumption that students’ life satisfaction and mood increase in late adolescence and that the increase differs among students.

### 3.3. Covariates of Change

In the next step we checked which of the considered covariates were either associated with the intercept or change factor of both life satisfaction and mood. First, every covariate was tested separately.

**Figure 1:**
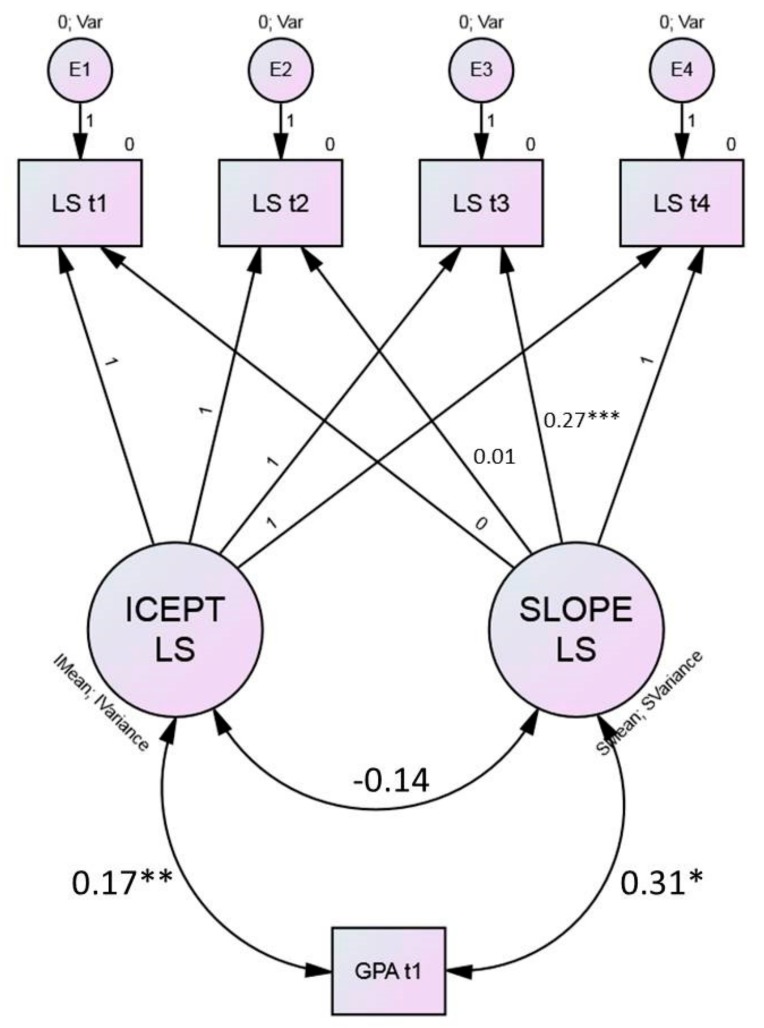
Sample figure for the latent growth curve model for life satisfaction (LS) with grade point average (GPA) measured at t1 as a moderator.

A sample model is illustrated in Figure 1 for GPA.

Table 4 shows the correlations of all covariates with the slope and intercept factors of both life satisfaction and mood.

**Figure 2:**
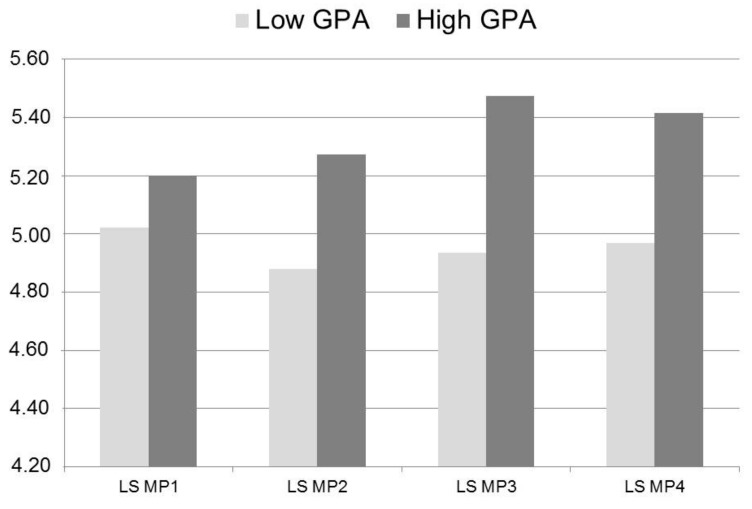
Mean scores in life satisfaction of students with above average and below average GPA at t1. LS = life satisfaction, GPA = grade point average.

All correlations with the intercept factors were in line with the correlations found between the covariates and the observed scale scores at t1 for life satisfaction and mood (see Table 1). Change in life satisfaction was positively associated with GPA at t1 and negatively with Extraversion. Students with better grades at t1 did not only have on average a higher life satisfaction at t1 but had also a greater increase in their life satisfaction from t1 to t4 as depicted in Figure 1. To compare better and worse performing students’ life satisfaction we conducted a median split. Students with a Grade Point Average (GPA) ≤ 4.09 were labelled “Low GPA” and those with a GPA > 4.09 as “High GPA” (see Figure 2).

**Figure 3:**
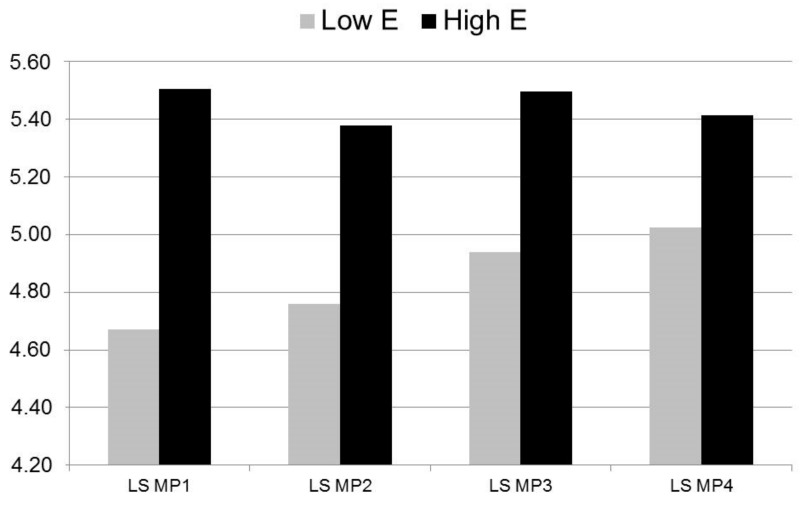
Mean scores in life satisfaction of students with above average and below average Extraversion at t1.

To illustrate the effect of extraversion on change in life satisfaction, students were categorized into two groups, below the median extraversion score (≤3.58; labelled introverts) and above (>3.58; labelled extraverts), via median split. Introverts reported lower life satisfaction at t1 but their life satisfaction increased more strongly from t1 to t4 than extraverts’ life satisfaction (see Figure 3). However, introverts’ life satisfaction was still significantly different from extraverts’ life satisfaction at t4 (F_1,243_ = 7.04, *p* = 0.009).

**Figure 4:**
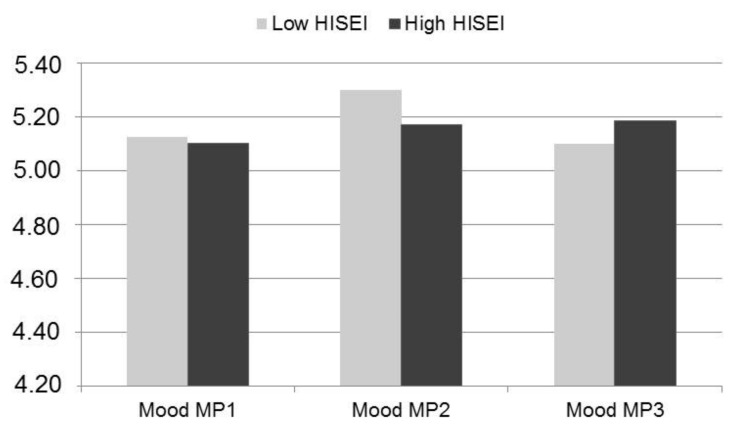
Mean scores in mood of students with above average and below average highest international socio-economic index of occupational status (HISEI)-scores at t1.

Change in mood was positively associated with students’ socio-economic background. Again, we performed a median split (“Low HISEI” hisei ≤ 54; “High HISEI” hisei > 54). Whereas students from low socio-economic households displayed a rather u-inverted change pattern in mood, students from families with a higher socio-economic status steadily increased in their reported mood (see Figure 4). However, both groups did not significantly differ from each other at any measurement point. Furthermore, it should be kept in mind that some students in the “Low HISEI” group had a higher HISEI-score than the average HISEI-score in a representative sample (see above).

The result pattern did not change when all variables were regressed on the mood and life satisfaction slope factor simultaneously with one exception: gender became a significant predictor of the life satisfaction slope factor (*β* = −0.45, *p* = 0.001) when controlling for the other covariates.

## 4. Discussion

The present study examined the SWB development of adolescents at the end of schooling and what variables might be related to interindividual differences in SWB but also with interindividual differences in intraindividual change. Interindividual differences in mood and life satisfaction were more strongly associated with personality traits (top-down factors) than with socio-demographic variables such as HISEI or GPA (bottom-up factors) replicating the pattern found for adults [107]. Both life satisfaction and mood increased from 11th to 13th Grade but there was substantial interindividual variation in that change. Among the investigated covariates we found GPA and extraversion to moderate the change in life satisfaction and HISEI to moderate the change in mood. Gender was tentatively associated with change in life satisfaction and extraversion with change in mood.

### 4.1. Cross-Sectional Correlations with SWB

Intelligence was not related to SWB at most measurement points. As in the present study, the association between intelligence and indicators of SWB seems to vary [60,62] but many studies with school students have also not found a correlation between the two variables, e.g., with happiness in 11th graders [24]. Rather, as the less able students tend to drop out during the last years of school, the significant association between intelligence and life satisfaction at measurement point 4 might indicate that there is a curvilinear association between intelligence and SWB. This should be investigated in further studies. Another interesting finding was that parents’ help with school tasks or how much parents value high attainment in school was not or only slightly associated with SWB. However, talking with parents was more consistently associated with SWB (t1–t2). The fact that at t4 none of the family variables were associated with SWB may coincide with an increase in the adolescents’ autonomy with respect to the family. Furthermore, the more consistent association with “Talking with parents about school” might indicate a process about how to improve adolescents’ SWB as intervention studies have demonstrated that talking to important others is a critical indicator of how relationships can increase individuals’ SWB [108]. Therefore, even though the association between SWB and “Talking with parents” was not large, it may indicate an important effect, because in the case of process variables small effects can also be impressive [109].

### 4.2. Developmental Changes in SWB

The overall positive change in life satisfaction and mood parallels those of a Finnish study that also found an increase in life satisfaction in Ninth to 11th Graders [14]. The present study replicates these findings in an older sample demonstrating that the positive trend in SWB continues after 11th Grade and extends these findings to the SWB component mood. Studies with younger students (e.g., Seventh–Ninth Grade; [15,19,110]) found a negative development in life satisfaction. Puberty is characterized by stages of low self-esteem, depression, temper, etc. [33], which are negatively associated with both mood and life satisfaction [12]. As most students up to ninth grades are experiencing puberty, the different stages of puberty might explain the decrease in life satisfaction up to Ninth Grade and the increase afterwards. When puberty ends, self-esteem and self-efficacy increase [111,112]. As these are important determinants of SWB [12], the increase in these important predictors of SWB might also lead to an increase in SWB, which might explain why youth in late adolescence are more content and happier with their lives than adolescents still in the middle of their puberty. However, this change in pattern varies between individuals as the variances of both slope factors were significant, which was also reported by Salmela-Aro and Tuominen-Soini [14]. Therefore, the interindividual change in both life satisfaction and mood varies between adolescents, which is better captured by LGM than by repeated measures ANOVA [102]. The present study is one of the first to investigate potential moderators of developmental changes in SWB.

### 4.3. Moderators of Developmental Changes in SWB

As described above, theories of SWB can broadly be subdivided into bottom-up and top-down theories [23]. Whereas bottom-up theories identify factors that relate to aspects that consistently affect SWB, such as socio-demographic factors, top-down theories focus on factors such as personality, that are related to aspects within a person and shape SWB by the way we experience our life and life events. In accordance with numerous studies with adults, personality accounted more for interindividual differences in SWB than bottom-up factors such as gender or socio-economic status [8,113]. Actually, gender and socio-economic status were not related to SWB or mood at any measurement point. However, the present study extends existing findings as it demonstrates that the socio-economic status of the family was positively related to interindividual differences in adolescents’ intraindividual changes in mood. If students were from families with a high SES, their mood increased more positively than those of students from less privileged families. Studies with adults [114] consistently demonstrated negative associations between SES and depression. As mood scales and depression are highly negatively correlated [115], it might well be that the onset of the association between SES and depression might be rooted in late adolescence. If the mood of students from socially advantaged families increases from late adolescence on, whereas it remains rather stable or even declines in socially disadvantaged youths, the gap between the two groups increases and thereby results in a positive relationship between mood/depression and SES in adults. One possible mechanism is that in late adolescence students from economically disadvantaged families start to worry more than their more privileged peers who can be more certain about economic and, perhaps, educational support from their parents. For example, Hoy, Tarter and Hoy [116] demonstrated that SES and academic optimism are related. However, this result should not be over-interpreted as SES was not significantly associated with mood or life satisfaction at any measurement point. Furthermore, the investigated sample was not representative with regards to its average SES, as it is common at the Gymnasium, the highest academic school track in Germany. Consequently, some students belonging to the below average SES group had a higher SES indicator than those found for the average SES in a representative sample. Further studies with representative samples should investigate whether they are able to replicate these results or if the relationship between SES and change in mood is curvilinear, i.e., that only those adolescents with a far above average SES display a more positive change in mood than their peers. Furthermore, interventions aiming at the emotional component of SWB should especially focus on socially disadvantaged young people.

Gender was not associated with life satisfaction or mood at any measurement point. This is in line with previous studies that also did not find an association between gender and SWB measures [32,117]. However, research on gender differences in SWB is very inconsistent. Batz and Tay [118] provide several explanations for the often found lack of gender differences and provide two categories with explanations for the lack of gender differences in SWB: “Return to Baseline” and “Basis of Evaluation”. For the latter, the proposed psychological processes are “In-Group Social Comparisons” and “Values”. For the former, the proposed psychological processes are adaption and habituation. Applied to school, it might well be that no gender differences are observed as female and male adolescents adopt equally well to school and other demands. Furthermore, especially in puberty, adolescents tend to compare themselves with same-sex peers [119]) so that any objective gender differences in variables influencing SWB, e.g., grades [12], might not result in gender differences in SWB. However, despite the lack of gender differences in SWB at single measurement points, we found a tentatively significant effect of gender on intraindividual change in life satisfaction. This result is in line with that reported by Salmela-Aro and Tuominen-Soini [14] who found a more positive change in life satisfaction for girls than for boys among slightly younger students. As their sample was larger than the present one, it may be that the present data did not have the power to detect the effect found by Salmela-Aro and Tuominen-Soini [14]. However, the gender difference in life satisfaction change became significant when controlling for the other covariates. Among the variables most highly correlated with life satisfaction, gender displayed the highest correlation with neuroticism. Consequently, neuroticism might have a negative impact on the otherwise positive change in life satisfaction for girls.

Among the personality variables, intelligence was neither associated with life satisfaction and mood at any measurement point nor with their change. This is in line with previous studies that also did not find an association between SWB and intelligence [50,62]. The present study extends these findings by showing that intelligence is additionally not associated with change in life satisfaction or mood. Consequently, the level and development of individuals’ SWB is independent of intelligence. In accordance with the meta-analytical results by Steel, Schmidt, and Shultz [46], life satisfaction and mood displayed medium to high correlations with neuroticism and extraversion. Therefore, the present study shows that the strong association between personality and SWB is already given in adolescence. The present study is the first that investigated if personality moderates the intraindividual change in SWB in adolescence. Extraversion, but not neuroticism, moderated the intraindividual change in life satisfaction and, tentatively, in mood. Although introverts’ SWB was significantly lower than extraverts’ at all measurement points, the gap between the two groups diminished from t1 to t4. Introverts might be more content with their lives and tentatively have better mood at the end of their school years, as behaviors associated with being extraverted (going to parties, having a lot of friends) may not be as important for youth at that stage as in earlier years. Therefore, the increased person-environment fit might lead to a higher SWB and thus to positive intraindividual changes of SWB in introverts [120]. However, introverts were still less content and happy with their lives as extraverts at the end of the study. Therefore, introverts might especially benefit from an intervention for enhancing SWB, however, if interventions are likely to work better with introverts than with extraverts is a different question. In the study by de Vibe conducted with medical and psychology students, extraversion did not moderate the effect of the SWB training [121].

Perceived parental support and values were not consistently or not at all associated with both indicators of SWB. Among the items “Talking with parents about school” (indicator of the quality of relationship) was most consistently associated with both life satisfaction and mood. Talking with parents about school is often considered as an indicator of the social resources available to a child [76]. However, substantive communication is also associated with SWB [122]. As school plays an important role in adolescents’ lives, talking about it might be expected to increase their SWB. However, talking with one’s parents about school was not associated with interindividual difference in intraindividual change in both components of SWB. Therefore, the individual development of SWB seems not to be influenced by talking with one’s parents about school in the investigated time range. Parents’ value of high attainment in school was less consistently associated with life satisfaction or mood. This result is in line with previous studies that failed to demonstrate an association between SWB and perceived parental academic pressure [123], a construct related to parents’ valuing of high scholastic attainment. The importance parents directly or indirectly put on school seems not to be highly related to students’ SWB, and, seems not to be a prerequisite for intraindividual change.

GPA was not only associated with life satisfaction at all measurement points but also significantly associated with the intraindividual change in life satisfaction. In line with prior studies, the bivariate correlations between life satisfaction and grades were rather small in size [49,124]. However, GPA was neither related to mood at all measurement points nor to its change [56]. Therefore, grades seem to be unrelated to mood but important for life satisfaction. From prior studies [56] we know that grades predict interindividual change in life satisfaction whereas the reciprocal effect is less consistent (some studies have found an effect of life satisfaction on interindividual change in grades: e.g., [125,126] however, others have not: e.g., [56]). The present study extends the debate on the relationship between academic achievement and life satisfaction by showing that grades do not only predict interindividual change but also intraindividual change in life satisfaction. Grades play a major role in students’ life, both in the present and in their future (especially in Germany; [127]) so that having better grades might be one prerequisite for becoming more content with one’s life (intraindividual changes) given an already higher intercept for students with better grades at t1. This effect of grades on intraindividual change seems to be larger than the effects of grades on interindividual change, which is rather small [56]. Given the important role that school plays in students’ life, schools should provide interventions to enhance students’ SWB and to buffer the effect that grades seem to have for intraindividual change in life satisfaction [128]. As interventions for enhancing SWB also increase school performance, schools and students would benefit in multiple ways [129].

### 4.4. Limitations and Outlook

The present study extends our knowledge of the development of SWB in many respects. However, it also suffers from some limitations. First, the investigated sample was not representative. Students from academic-track schools in Germany are more intelligent [130], have higher school performance [124] and are more socially advantaged [86]. Furthermore, more girls attend this kind of school. As boys are selected more strictly for academic-track schools they tend to be more intelligent, as was found in the present sample. Both the restriction in range which leads to a diminished variance in the sample, the overrepresentation of girls, and just looking at the medium and upper range of the distribution might have distorted the observed results. Therefore, future studies should replicate the reported results in representative samples. Second, we only investigated SWB longitudinally. Even though we gained some valuable insights into adolescents’ intraindividual development of SWB, future studies should not only assess SWB longitudinally but also some of the observed covariates such as personality. Additionally, analyses could be performed on predictors of the interindividual change of both SWB and other variables and the direction of these effects. In this context, it might be especially interesting to focus on different familial variables such as the attachment to parents, parenting behaviors, or parenting styles [76,131,132]. Furthermore, it might be promising to examine when the influence of familial variables decreases and the influence of peer variables increase throughout development in late adolescence. Finally, we only investigated students at the end of schooling. At this time, attending a school is not mandatory any more. As students in this age range voluntarily attend school in Germany, their SWB might have already been enhanced. The self-determination theory postulates that autonomous decisions increase SWB [133]. Further studies should investigate whether the same pattern of results can be found in samples of the same age when schooling is still mandatory.

## 5. Conclusions

The present study investigated intraindividual change in SWB and factors with which it is associated. In late adolescents (16–19 years) both mood and life satisfaction increased. However, there were substantial individual differences in that intraindividual change. Interindividual differences in intraindividual changes in mood were related to students’ social background whereas interindividual differences in intraindividual change in life satisfaction were related to extraversion and grades. Therefore, interventions aiming at increasing mood should especially focus on socially disadvantaged students. Interventions aiming at increasing life satisfaction should especially focus on introverted students. Even though introverts’ intraindividual change in life satisfaction was stronger than that of extraverted students, introverts’ life satisfaction was still lower than extraverts’ at the end of the study. Interventions aiming at both components of SWB should especially focus on poor performing students as they run the risk that their SWB does not increase as strongly as those of better performing students.

## Figures and Tables

**Table 1:** Overview of when each variable was assessed.

Variable	t1	t2	t3	t4
Life satisfaction	x	x	x	x
Mood		x	x	x
Covariates	x			

**Table 2:** Means (*M*), standard deviations (*SD*), internal consistencies (*α*), and intercorrelations among all predictors and criteria.

	Descriptives		Intercorrelations															
	*M*	*SD*	*(1)*	*(2)*	*(3)*	*(4)*	*(5)*	*(6)*	*(7)*	*(8)*	*(9)*	*(10)*	*(11)*	*(12)*	*(13)*	*(14)*	*(15)*	*(16)*
(1) Life satisfaction t1	5.11	1.07	1	-	-	-	-	-	-	-	-	-	-	-	-	-	-	-
(2) Life satisfaction t2	5.11	1.15	0.65 **	1	-	-	-	-	-	-	-	-	-	-	-	-	-	-
(3) Life satisfaction t3	5.26	1.04	0.64 **	0.60 **	1	-	-	-	-	-	-	-	-	-	-	-	-	-
(4) Life satisfaction t4	5.30	1.15	0.55 **	0.62 **	0.65 **	1	-	-	-	-	-	-	-	-	-	-	-	-
(5) Mood t2	5.12	1.19	0.55 **	0.71 **	0.49 **	0.45 **	1	-	-	-	-	-	-	-	-	-	-	-
(6) Mood t3	5.26	1.14	0.54 **	0.47 **	0.75 **	0.50 **	0.61 **	1	-	-	-	-	-	-	-	-	-	-
(7) Mood t4	5.20	1.25	0.49 **	0.53 **	0.52 **	0.77 **	0.59 **	0.64 **	1	-	-	-	-	-	-	-	-	-
(8) Gender	1.50	0.50	0.08	0.02	−0.05	0.01	−0.02	−0.06	−0.01	1	-	-	-	-	-	-	-	-
(9) HISEI	56.52	12.72	0.06	0.06	0.03	0.08	−0.03	0.00	0.07	0.05	1	-	-	-	-	-	-	-
(10) Intelligence	109.90	16.93	0.03	0.05	0.08	0.17 **	0.01	0.03	0.06	0.33 **	0.05	1	-	-	-	-	-	-
(11) Neuroticism	2.75	0.63	−0.46 **	−0.39 **	−0.37 **	−0.37 **	−0.40 **	−0.34 **	−0.40 **	−0.32 **	0.00	−0.12 *	1	-	-	-	-	-
(12) Extraversion	3.54	0.54	0.46 **	0.36 **	0.27 **	0.28 **	0.56 **	0.46 **	0.48 **	−0.09	−0.08	−0.07	−0.30 **	1		-	-	-
(13) Support	1.96	0.94	0.07	0.04	−0.03	0.05	0.11	0.00	0.13 *	−0.06	0.16 **	−0.12 *	0.13 **	0.11 *	1	-	-	-
(14) Quality of relationship	3.93	0.85	0.18 **	0.20 **	0.05	0.12	0.11 *	0.11	0.15 *	−0.10 *	0.18 **	−0.05	−0.07	0.16 **	0.21 **	1	-	-
(15) Parental scholastic values	3.91	0.69	0.10 *	0.07	0.06	0.12	0.03	0.02	0.03	0.08	0.12 *	0.12 *	−0.09	0.00	0.01	0.20 **	1	-
(16) GPA	4.08	0.59	0.10 *	0.18 **	0.27 **	0.23 **	0.03	−0.01	0.06	−0.03	0.17 **	0.32 **	−0.08	−0.07	−0.08	0.17 **	0.18 **	1

Notes: *N* = 289–476, * *p* < 0.05, ** *p* < 0.01, HISEI = highest international socio-economic index of occupational status, GPA = grade point average.

**Table 3:** Factor Loadings and Standard Errors (SE) of the Slope Factor for the General Development Model.

Factor	Coefficient Estimations	t1	t2	t3	t4
Life satisfaction	Estimate of factor loading	0	−0.16	0.68 ***	1
	SE		0.22	0.20	
Mood	Estimate of factor loading		0	0.78 ***	1
	SE			0.23	

Note. *** *p* ≤ 0.001. Variables printed in grey are not significant.

**Table 4:** Model fit and slopes and intercept factors’ correlation with all covariates considered separately.

Variable	Life Satisfaction Model Fit			LifeSatisfaction		Mood Model Fit			Mood	
	*χ* *^2^*	CFI	RMSEA	Intercept	Slope	*χ* *^2^*	CFI	RMSEA	Intercept	Slope
Gender	20.07	0.98	0.06	0.02	−0.20 ^#^	10.35	0.98	0.07	−0.03	−0.004
HISEI	15.91	0.99	0.05	0.07	0.01	10.25	0.98	0.07	−0.06	0.28 *
Intelligence	15.37	0.99	0.04	0.05	0.20	9.71	0.98	0.07	−0.004	0.12
Neuroticism	20.22	0.98	0.06	−0.52 ***	0.10	4.77	1.00	0.04	−0.46 ***	0.00
Extraversion	23.64	0.98	0.06	0.53 ***	−0.36 **	9.19	0.99	0.07	0.69 ***	−0.24 ^#^
Support	15.89	0.99	0.05	0.08	−0.03	12.71	0.97	0.08	0.10	0.03
Quality	17.18	0.99	0.05	0.22 ***	−0.20	9.99	0.98	0.07	0.13 *	0.09
Parental scholastic value	17.77	0.98	0.05	0.09	0.02	9.83	0.98	0.07	0.02	0.03
GPA	19.58	0.98	0.06	0.17 **	0.31 *	14.09	0.96	0.09	0.04	−0.01

Note. CFI = Comparative Cit Index, RMSEA = Root-Mean Square Error of Approximation, models life satisfaction: df = 8, models mood: df = 3; ^#^ *p* ≤ 0.10 * *p* ≤ 0.05, ** *p* ≤ 0.01,*** *p* ≤ 0.001.
